# Clinical improvement of pemphigus-like mucocutaneous disease in a patient with CLL following initiation of zanubrutinib: a case report

**DOI:** 10.3389/fimmu.2026.1876086

**Published:** 2026-09-15

**Authors:** Tianyu Li, Chengbin Lu, Tong Kang, Ying Tu, Dongjie Sun

**Affiliations:** 1 Department of Dermatology, The First Affiliated Hospital of Kunming Medical University, Yunnan, Kunming, China; 2 The Second Xiangya Hospital, Central South University, Changsha, Hunan, China

**Keywords:** autoimmune bullous disease, B-cell chronic lymphocytic leukemia, BTK inhibitor, case report, paraneoplastic pemphigus, zanubrutinib

## Abstract

Pemphigus is an autoimmune bullous disease. PNP/PAMS represents a rare subtype of autoimmune bullous disease, and typically associated with an underlying neoplasm, particularly lymphoproliferative disorders. Current management of PNP primarily involves glucocorticoids, immunosuppressants, and rituximab. This article reports a 67-year-old male who presented with generalized erythema, blisters, and oral erosions with pain for one month. Initially diagnosed with pemphigus, he received methylprednisolone treatment, which led to slight improvement in skin lesions but minimal resolution of oral ulcers. Based on the available clinical, histopathological, immunofluorescence, and serological findings, the patient was initially diagnosed with pemphigus vulgaris in the setting of CLL. However, the refractory oral mucositis, dual positivity for Dsg1 and Dsg3, and the underlying B-cell malignancy raised the concern for paraneoplastic pemphigus (PNP). Given that PNP-specific tests—including anti-plakin antibodies, indirect immunofluorescence on rat bladder, and immunoblotting—were not performed, a definitive diagnosis of PNP could not be established. Therefore, the condition was managed as “suspected CLL-associated PNP” Following the initiation of zanubrutinib in combination with ongoing corticosteroid therapy and CLL treatment, significant improvement of oral erosions was observed. In this case, the clinical improvement may be potentially related to zanubrutinib, but the concurrent corticosteroid therapy and the control of CLL likely contributed as well. If zanubrutinib did play a role, the mechanism might involve enhanced disease control or immune modulation. However, the current evidence is insufficient to draw a firm conclusion, and its true efficacy needs to be evaluated in further studies.

## Introduction

Pemphigus is a rare autoimmune blistering disorder characterized by vesicles, bullae, and erosions involving the skin and mucous membranes, often accompanied by pruritus and pain, substantially impairing quality of life (1–3). Paraneoplastic pemphigus (PNP) represents an exceedingly rare subtype of autoimmune bullous disease (4, 5). A subset of PNP cases is closely associated with hematologic malignancies, particularly chronic lymphocytic leukemia (CLL) (6–8). In recent years, attention to PNP especially malignancy-associated PNP has increased; However, its pathogenesis remains incompletely understood (9). Current evidence suggests that tumor-triggered autoimmune responses directed against desmosomal and hemidesmosomal proteins play a central role in disease development. Management strategies primarily focus on effective treatment of the underlying neoplasm, alongside systemic corticosteroids, immunosuppressive agents, and biologic therapies such as rituximab, which have shown clinical benefit. Nevertheless, therapeutic options for B- cell-driven PNP remain limited (10). Zanubrutinib, a second-generation Bruton tyrosine kinase inhibitor (BTKi), has been approved for the treatment of CLL and small lymphocytic lymphoma (SLL) (11–13). Given that the BTK signaling pathway is pivotal in B-cell activation, proliferation, and autoantibody production, BTK inhibition may offer a potential approach to modulating autoimmune responses (14–16). Although zanubrutinib is increasingly used in CLL, clinical experience with its application in CLL-associated PNP remains scarce (17, 18). Here, We report a patient with CLL and suspected CLL-associated PNP whose skin and oral mucosal lesions improved in the setting of combination therapy with corticosteroids and zanubrutinib.

## Case presentation

A 67-year-old man was admitted with a 1-month history of generalized erythema, blisters, and erosions accompanied by pain, and a 2-week history of worsening oral mucosal erosions. More than 1 month prior to admission, he developed erythema and blisters on the trunk and proximal extremities without an identifiable trigger. Some lesions ruptured, forming erosions associated with significant pain. The Nikolsky sign was positive, and approximately 18% of the body surface area (BSA) was involved. One week after disease onset, multiple erosions developed on the buccal mucosa, lateral tongue, and gingiva, accompanied by severe pain during eating, with a visual analog scale (VAS) score of 7. There was no involvement of the conjunctival, genital, or respiratory mucosa. The patient had been previously healthy, with no history of hematologic disorders, malignancy, drug allergy, or chronic infection. He reported no recent medication use prior to the onset and denied B symptoms associated with chronic lymphocytic leukemia (CLL), such as fever, weight loss, or night sweats. There was no family history of autoimmune blistering diseases or hematologic malignancies, and no relevant psychosocial stressors were identified. On physical examination, the patient was in fair general condition. Multiple enlarged superficial lymph nodes were palpable, most prominently in the cervical, axillary, and inguinal regions, with the largest measuring approximately 3.2 × 1.8 cm. These nodes were firm, non-tender, and mobile. Dermatologic examination revealed scattered erythema, blisters, and erosions on the trunk and extremities, some with crusting. Multiple erosions and shallow ulcers were observed on the buccal mucosa and lateral tongue (**Figure 1**).

**Figure 1:**
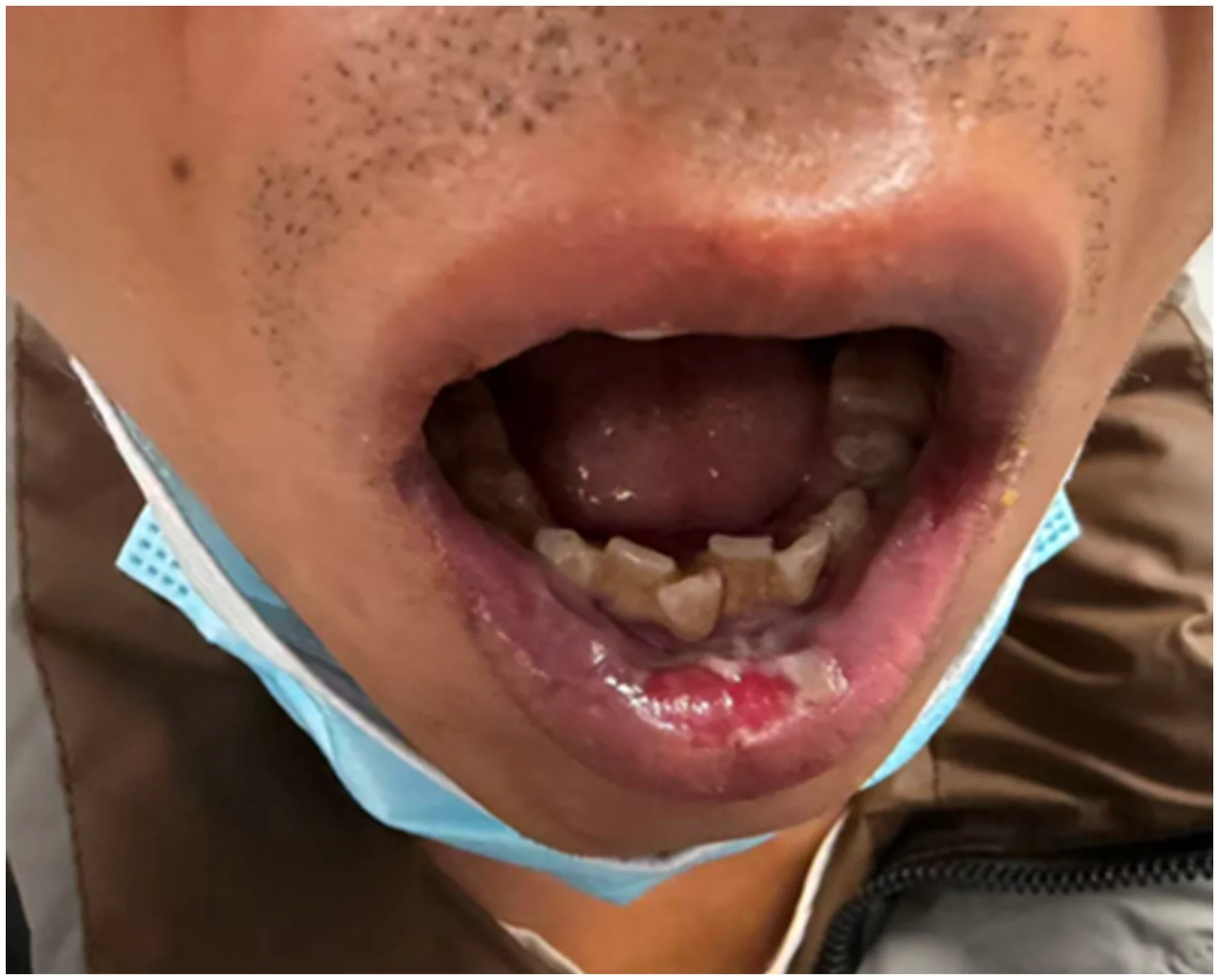
Physical examination on admission: oral mucosal erosion.

Skin biopsy demonstrated suprabasal acantholysis with intraepidermal blister formation. Acantholytic cells were present within the blister cavity. In the superficial dermis, perivascular infiltration of lymphocytes and histiocytes was observed. These findings were consistent with pemphigus vulgaris (**Figure 2**). Direct immunofluorescence revealed intercellular deposition of IgG (++) and C3 (++) in a reticular pattern within the epidermis (**Figure 3**).

**Figure 2:**
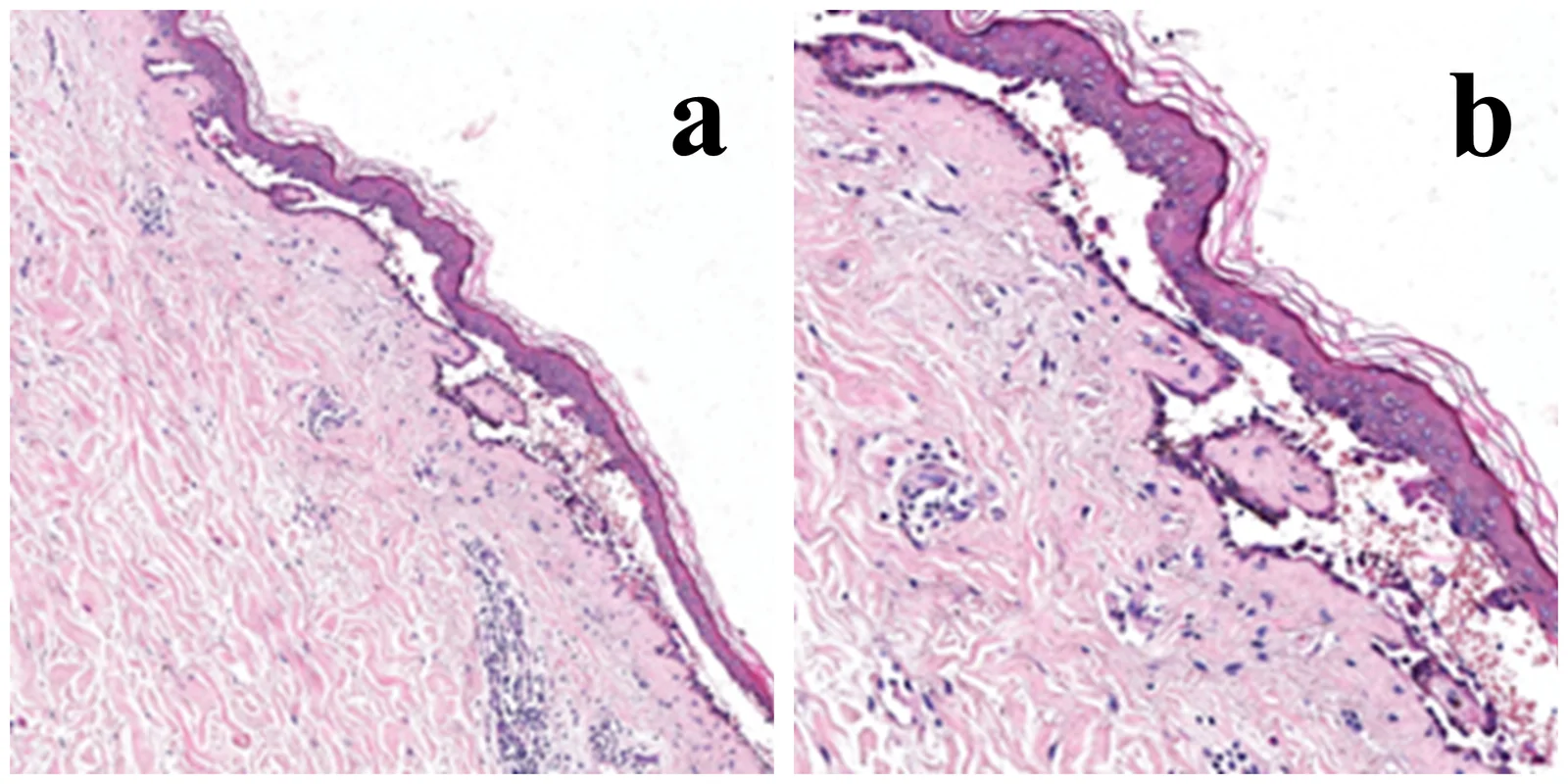
Histopathological findings of the skin lesion. **(a)** intraepidermal acantholysis and blister formation; **(b)** perivascular lymphocytic and histiocytic infiltration in the superficial dermis. Hematoxylin and eosin staining, original magnification ×20.

**Figure 3:**
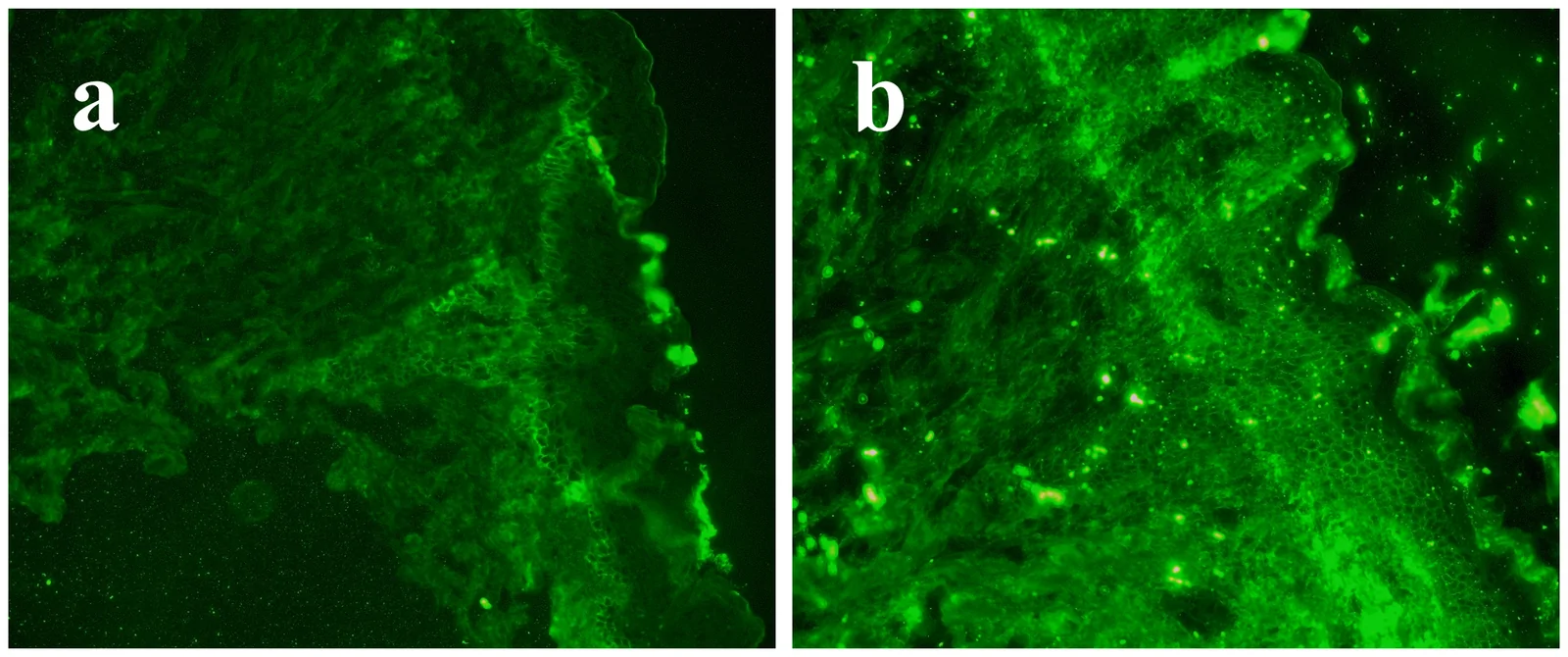
Direct immunofluorescence findings. **(a, b)** Intercellular reticular deposition of IgG and C3 within epidermal tissue.

Serologic testing showed markedly elevated levels of anti-desmoglein 1 (Dsg1) and anti-desmoglein 3 (Dsg3) antibodies (>150 U/mL). Anti-BP180 and anti-BP230 antibodies were both <20 RU/mL. Peripheral blood smear demonstrated an increased proportion of lymphocytes, occasional atypical lymphocytes, and frequent smudge cells. Bone marrow cytologic examination revealed a markedly increased proportion of lymphocytes, suggesting a lymphoproliferative disorder (**Table 1**).

**Table 1:** Bone marrow cytology results.

Cell lineage	Cell type	Percentage (%)	Reference range (%)
Granulocytic series	Promyelocytes	1.0	1.0–5.0
	Neutrophilic myelocytes	10.0	5.0–20.0
	Neutrophilic metamyelocytes	11.0	5.0–15.0
	Band neutrophils	14.0	10.0–30.0
	Segmented neutrophils	10.0	5.0–20.0
Erythroid series	Proerythroblasts	1.0	1.0–3.0
	Basophilic erythroblasts	5.0	5.0–20.0
	Polychromatic erythroblasts	9.0	5.0–15.0
Lymphoid series	Lymphocytes	35.0 ↑	10.0–30.0
Monocytic series	Monocytes	1.0	1.0–5.0
Plasma cell series	Plasma cells	1.0	0–2.0
Other cells	Histiocytes	2.0	0–2.0
Myeloid-to-erythroid ratio	–	3.07:1	2.00–5.00:1

The initial complete blood count showed WBC 13.81×10^9^/L, absolute lymphocyte count 9.90×10^9^/L, lymphocyte percentage 71.7%, hemoglobin 134 g/L, and platelets 230×10^9^/L. Peripheral blood flow cytometry identified an abnormal B-lymphocyte population accounting for approximately 34.6% of nucleated cells (CD5^+^, CD10^−^), with small forward scatter, expressing CD23, weakly expressing CD20 and CD22, partially expressing CD200, and negative for FMC7, sIgM, and CD79b. The immunophenotype was suggestive of a B-cell chronic lymphoproliferative disorder (B-CLPD), most likely CLL. The absolute clonal B-cell count was not provided in the flow cytometry report.

Bone marrow aspirate showed 35% lymphocytes, 1% promyelocytes, 10% neutrophilic myelocytes, 11% neutrophilic metamyelocytes, 14% neutrophilic band forms, 10% neutrophilic segmented forms, 1% proerythroblasts, 5% basophilic erythroblasts, 9% polychromatic erythroblasts, with a myeloid-to-erythroid ratio of 3.07:1. Plasma cells accounted for 1% and histiocytes for 2%. No increase in blasts was observed. Bone marrow biopsy demonstrated scattered or small clusters of small lymphoid cells; immunohistochemistry showed CD20 positivity and scant CD3 positivity.

Chest CT performed on November 14, 2024, revealed scattered tiny and small pulmonary nodules (Lung-RADS category 2), scattered linear opacities in both lungs, several slightly enlarged mediastinal lymph nodes, bilateral axillary lymphadenopathy, aortic tortuosity with minor calcification of the aorta and coronary arteries, and a possible old fracture of the anterior left eighth rib.

After treatment, the complete blood count showed WBC 4.55×10^9^/L, absolute lymphocyte count 2.59×10^9^/L, lymphocyte percentage 56.9%, hemoglobin 155 g/L, and platelets 201×10^9^/L. FISH for del(17p)/TP53 and IGHV mutational analysis were not performed at our institution. The absolute clonal B-cell count could not be obtained as it was not provided in the flow cytometry report.

Ultrasonography of the cervical, axillary, and inguinal regions revealed multiple enlarged lymph nodes in the bilateral submandibular areas, along the sternocleidomastoid muscles, and in the supraclavicular and infraclavicular fossae. The lymph nodes had well-defined borders; the largest measured approximately 2.4 × 1.0 cm on the right and 3.2 × 1.8 cm on the left. The corticomedullary differentiation was indistinct, more pronounced on the left. Multiple enlarged lymph nodes were also detected in both axillae, with the largest measuring approximately 2.4 × 1.1 cm on the left and 1.8 × 1.0 cm on the right. Corticomedullary differentiation was again poorly defined. Color Doppler flow imaging (CDFI) and color Doppler energy (CDE) demonstrated a hilar vascular pattern. Similarly, multiple enlarged lymph nodes were observed in both inguinal regions, with the largest measuring approximately 1.8 × 1.1 cm on the right and 1.4 × 1.0 cm on the left. These nodes also exhibited poorly defined corticomedullary differentiation and a hilar vascular pattern on CDFI and CDE. Biopsy of the largest left inguinal lymph node was recommended. Biopsy of a left cervical lymph node revealed a proliferative lymphoid lesion, and lymphoma could not be excluded.

Based on peripheral blood flow cytometry, bone marrow aspiration and biopsy, and imaging findings, a diagnosis of **B-cell chronic lymphocytic leukemia (CLL)** was considered most likely. The patient had no anemia (Hb 134 g/L) or thrombocytopenia (PLT 230×10^9^/L) and was staged as **Rai stage I/Binet stage B**. Small lymphocytic lymphoma (SLL) was considered unlikely due to documented bone marrow and peripheral blood involvement. Monoclonal B-cell lymphocytosis (MBL) was also considered unlikely given the presence of lymphadenopathy, bone marrow involvement, and an elevated absolute lymphocyte count. The diagnosis was confirmed by the attending hematologist.

According to the 2025 Chinese Expert Consensus on the Diagnosis and Treatment of Paraneoplastic Pemphigus, the patient’s condition fulfilled the following criteria for a clinical diagnosis of suspected CLL-associated PNP. Typical clinical presentation: Refractory oral mucosal erosions accompanied by pemphigus-like skin lesions. The cutaneous lesions showed partial improvement with corticosteroids, while the mucosal involvement persisted a feature that raises suspicion for PNP. Histopathology: Suprabasal acantholysis with intraepidermal blister formation, and perivascular lymphohistiocytic infiltration in the superficial dermis. Direct immunofluorescence (DIF): Intercellular reticular deposition of IgG and C3 (intercellular fluorescence). Serology: Both anti-desmoglein 1 (Dsg1) and anti-desmoglein 3 (Dsg3) antibodies were strongly positive (>150 U/mL), while anti-BP180 and anti-BP230 antibodies were negative. Underlying neoplasm: Confirmed diagnosis of B-cell chronic lymphocytic leukemia (CLL), the lymphoproliferative disorder most commonly associated with PNP.

It should be noted that the histopathology report did not describe typical interface dermatitis or dyskeratotic cells, and DIF only reported intercellular reticular deposition of IgG and C3 without mentioning linear deposition along the basement membrane zone. Nevertheless, the five core findings listed above fulfill the consensus-recommended criteria for a clinical diagnosis. Due to limited testing availability, anti-plakin antibodies were not assessed, and differentiation from pemphigus vulgaris coexisting with CLL remains necessary. The diagnosis made here is therefore a clinical one, and its limitations are further addressed in the Discussion. Indirect immunofluorescence on monkey esophagus, anti-desmocollin, and anti-envoplakin/anti-periplakin antibody testing were not performed owing to local testing limitations.

At initial presentation, the patient was diagnosed with pemphigus vulgaris and treated with intravenous methylprednisolone sodium succinate (60 mg daily). After 2 weeks, cutaneous lesions on the trunk and lower extremities gradually improved; however, oral lesions showed minimal response. The patient was subsequently maintained on oral methylprednisolone (32 mg in the morning and 16 mg in the evening) as an outpatient. After the hematologic work-up was completed and CLL was confirmed, a working diagnosis of suspected CLL-associated PNP was made. On November 28, 2024, CLL-directed therapy was initiated: the methylprednisolone dose was adjusted to 24 mg daily, and zanubrutinib (160 mg twice daily) was started as the first targeted therapy directed at the CLL clone. After 4 months of combined therapy, the patient exhibited substantial improvement in oral erosions and ulcers, with the visual analog scale (VAS) pain score decreasing to 2. The cumulative corticosteroid dose could not be calculated from the available records. Calcium and vitamin D were administered during corticosteroid therapy. No documentation of proton pump inhibitors or other prophylactic measures was found in the medical records.

Corticosteroid-associated complications were identified: in April 2025, vitamin deficiency was diagnosed; in July 2025, osteoporosis was documented. in August 25, 2025, endocrinology evaluation confirmed osteoporosis, prediabetes, and vitamin deficiency disease, and vitamin D with calcium carbonate was initiated. No fractures or severe infections were recorded. For a mild intermittent recurrence of cutaneous lesions while on zanubrutinib, the methylprednisolone dose was adjusted to 28 mg daily.

At the last follow-up in August 2025, no active erythema or erosive lesions was observed on the scalp, skin, or oral mucosa (**Figure 4**). Post‑treatment evaluation showed significant improvement of the oral mucosal lesions: pain had largely resolved, oral intake was normal, the total erosion area had decreased by more than 50%, and no new oral lesions were observed. In keeping with common clinical practice for pemphigusspectrum diseases, this was considered a partial response (defined as observable healing of erosions without complete re‑epithelialization). Because a standardized scoring system was not applied at baseline, this assessment is based on descriptive clinical records. Repeat laboratory testing showed normalization of the white blood cell count and improvement in the lymphocyte percentage. Overall, improvement was observed during combination therapy with corticosteroids and zanubrutinib. Serologic testing revealed a Dsg1 level of 11.73 RU/mL (normalized), while Dsg3 remained >400 RU/mL; anti-BP180 and anti-BP230 antibodies were both <20 RU/mL. Baseline testing on November 15, 2024, was performed by ELISA using a commercial kit (Hunan Xieguang Biotechnology Co., Ltd., China). According to the manufacturer’s instructions, the positivity cutoff is ≥20 RU/mL, which was established based on validation with no fewer than 120 clinical samples. Both anti-Dsg1 and anti-Dsg3 were reported as >150 U/mL; the laboratory report did not provide information on further dilution or exact titers. Anti-BP180 and anti-BP230 were both <2 RU/mL. Follow-up testing in August 2025 was performed using a magnetic particle chemiluminescence immunoassay. Anti-Dsg1 had decreased to 11.73 RU/mL, whereas anti-Dsg3 remained >400 RU/mL; no dilution information was provided. Of note, the two measurements were performed using different assay methods, which limits direct quantitative comparison between the baseline and follow-up values. These serological results should not be interpreted as direct evidence for the diagnosis of PNP, nor can they be attributed to any specific drug mechanism. The persistently elevated anti-Dsg3 level despite mucosal improvement may reflect ongoing autoreactive B-cell activity that lags behind the clinical response, although its precise clinical significance remains unclear.

**Figure 4:**
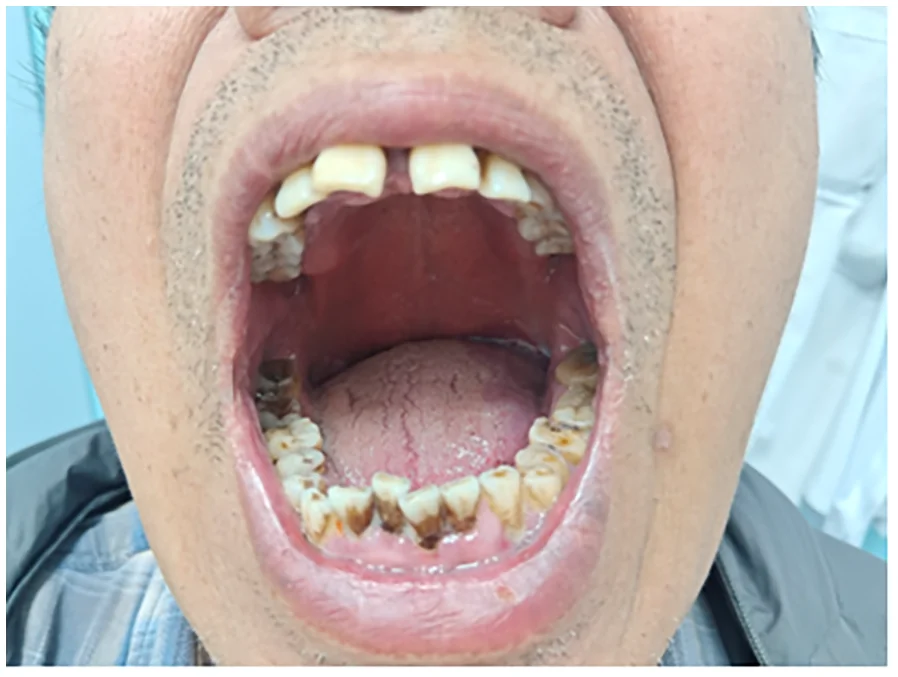
Clinical outcome after treatment: marked improvement of oral mucosal erosions.

With respect to respiratory involvement, the patient reported occasional cough but denied dyspnea, wheezing, chest tightness, or hypoxemia throughout the treatment course. Chest CT performed on November 14, 2024, revealed scattered tiny and small pulmonary nodules (Lung-RADS category 2), scattered linear opacities in both lungs, several slightly enlarged mediastinal lymph nodes, and bilateral axillary lymphadenopathy. No typical imaging features of bronchiolitis obliterans—such as air trapping or mosaic attenuation—were observed. Given the absence of significant respiratory symptoms and the low clinical suspicion for bronchiolitis obliterans at the time, pulmonary function testing was not pursued. Nevertheless, the potential risk of PNP-associated bronchiolitis obliterans warrants continued vigilance, and regular respiratory surveillance during follow-up is recommended.

### Patient perspective

“At the beginning, the most unbearable symptom was the severe sores in my mouth, along with blisters all over my body. Even swallowing saliva felt like being pricked by needles, and I could hardly eat anything. The pain kept me awake at night. After the diagnosis linked it to a blood disorder and Zanubrutinib was added, I noticed gradual improvement within about a month. The pain in my mouth lessened, and I was eventually able to eat soft foods again. Later, the oral erosions almost completely healed, and no new skin lesions appeared. Now I can eat and sleep normally, and my life has finally returned to normal”.

### Timeline of diagnosis and treatment

To provide a clear overview of the clinical course, a timeline of diagnosis and treatment is presented in **Table 2**. It is worth noting that despite persistently elevated Dsg3 antibody levels, the patient’s oral symptoms improved substantially, suggesting a discordance between serologic markers and clinical manifestations. Continued long-term monitoring is warranted due to the potential risk of relapse.

**Table 2:** Timeline of diagnosis and treatment.

Time point	Major clinical manifestations	Key findings	Treatment	Outcome
At admission (November 14, 2024)	Generalized erythema, blisters, multiple oral erosions; pain on eating (VAS 7); positive Nikolsky sign	Dsg1/Dsg3 >150 U/mL; histopathology and direct immunofluorescence consistent with pemphigus	Intravenous methylprednisolone 60 mg	Partial improvement in skin lesions; minimal improvement in oral erosions
2 weeks after treatment	Resolution of trunk and extremity lesions; persistent oral erosions (VAS 6)	No repeat evaluation performed	Oral methylprednisolone 32 mg (morning) + 16 mg (evening)	Discharged with regular follow-up
(November 28, 2024)	Persistent oral erosions; no new skin lesions	Bone marrow, lymph node biopsy, and flow cytometry supported diagnosis of CLL	Oral methylprednisolone 24 mg/day; addition of zanubrutinib 160 mg twice daily	Initiation of targeted therapy combined with corticosteroids
4 months after initiation of zanubrutinib	Substantial improvement in oral erosions; pain reduced (VAS 2); no new oral lesions	No repeat antibody or bone marrow testing	Continued zanubrutinib; methylprednisolone adjusted to 28 mg/day	Sustained clinical improvement
August 2025 (10months after zanubrutinib)	Oral erosions substantially improved (partial response); no active skin lesions	Normalized white blood cell count; Dsg1 11.73 RU/mL (normal); Dsg3 >400 RU/mL	Maintenance therapy with zanubrutinib plus low-dose corticosteroids	Partial response; ongoing follow-up

## Discussion

Paraneoplastic pemphigus (PNP) is a rare autoimmune blistering disease for which no standardized curative therapy exists, largely owing to its incompletely understood pathogenesis (5, 19). In recent years, the expert consensus or guidelines of many countries recommend glucocorticoids and immunosuppressants as the first-line treatment for PNP, with treatment of the underlying neoplasm considered central to disease management (4, 20). In cases associated with solid tumors, surgical resection of the primary tumor combined with short- to intermediate-term corticosteroid therapy often results in meaningful improvement of cutaneous lesions. However, when the underlying tumor is unresectable or cannot be adequately controlled, a therapeutic dilemma arises. The use of corticosteroids and immunosuppressants may compromise tumor control or even promote disease progression, potentially exacerbating PNP manifestations (6, 7, 21). Accordingly, current clinical practice emphasizes aggressive management of the underlying malignancy. On this basis, the European Academy of Dermatology and Venereology (EADV) recommends systemic corticosteroids in combination with immunosuppressive agents once the primary tumor is adequately controlled (20). Nevertheless, clinical outcomes remain suboptimal, underscoring the need for more effective therapeutic strategies.

In an analysis of 144 patients with PNP, Ouedraogo et al. reported that the majority of cases were associated with chronic lymphocytic leukemia (CLL) or non-Hodgkin lymphoma, far exceeding other tumor types (22). Because hematologic malignancies are not amenable to surgical resection, management of secondary PNP in this setting is particularly challenging. Zanubrutinib is an orally administered, highly selective, second-generation Bruton tyrosine kinase inhibitor (BTKi). By irreversibly binding to BTK, it reduces kinase activity and inhibits downstream signaling pathways critical for B-cell proliferation and differentiation. It has been approved for the treatment of CLL/small lymphocytic lymphoma (SLL) and has demonstrated favorable efficacy and safety in multiple clinical studies (11, 12). Previous case reports have shown that the first-generation BTKi ibrutinib can lead to significant improvement in cutaneous and mucosal lesions in patients with CLL-associated PNP, suggesting that BTK pathway inhibition may represent a promising therapeutic target (7). Given the central role of BTK signaling in B-cell activation, proliferation, and autoantibody production, BTK inhibitors have attracted increasing attention in the treatment of autoimmune diseases. BTK inhibitors suppress B-cell proliferation by blocking B-cell receptor signaling, and their potential has been explored in autoimmune conditions such as rheumatoid arthritis and systemic lupus erythematosus. Additionally, BTKis have shown therapeutic potential in systemic sclerosis, pemphigus vulgaris, and allergic diseases (23–25). In CLL-associated PNP/PAMS, however, BTK inhibitors may act through dual mechanisms—antitumor activity and direct immunomodulation—although current evidence is limited to case reports. The clinical contribution of each mechanism remains difficult to distinguish, and any potential benefit requires confirmation in further studies.

Reports on the use of zanubrutinib in CLL-associated PNP remain limited. In the present case, the patient was admitted with a 1-month history of generalized erythema, blisters, and erosions with pain. Initial evaluation was suggestive of pemphigus vulgaris; however, the subsequent emergence of refractory oral involvement and detection of CLL raised concern for PNP, and the working diagnosis was revised to suspected CLL-associated PNP. However, given the presence of generalized lymphadenopathy and abnormal hematologic findings, further evaluation including lymph node biopsy and bone marrow examination led to a diagnosis of B-cell-type CLL. In conjunction with histopathology, direct immunofluorescence, and serologic testing, a diagnosis of suspected CLL-associated PNP was made. However, several alternative diagnoses were considered in this patient. Pemphigus vulgaris (PV) co-existing with CLL is the most important differential. Although the refractory oral involvement and dual Dsg1/Dsg3 positivity raise suspicion for PNP, PV in the setting of CLL cannot be ruled out without testing for anti-plakin antibodies or indirect immunofluorescence on rat bladder. Drug-induced pemphigus was thought to be unlikely after a thorough medication review. Paraneoplastic autoimmune multiorgan syndrome (PAMS) overlaps significantly with PNP and could account for the mucocutaneous picture; however, the absence of pulmonary involvement and confirmatory serology precludes its confirmation. Lichenoid/mucosal interface dermatitis was considered but the histopathology did not show typical lichenoid changes. Other autoimmune blistering disorders, including bullous pemphigoid and linear IgA disease, were excluded by the negative anti-BP180/BP230 antibodies and the DIF pattern. Given the diagnostic uncertainty, the case was managed as “suspected CLL-associated PNP”.

Although systemic corticosteroid therapy led to significant improvement in cutaneous lesions, oral ulcers persisted. Following the initiation of zanubrutinib after confirmation of CLL, both the hematologic parameters and the oral mucosal lesions improved; apart from a mild cutaneous recurrence that responded to a corticosteroid dose adjustment, no new lesions observed during follow-up. However, because this patient had suspected CLL-associated PNP, it remains unclear whether the observed clinical benefit was primarily due to effective treatment of the underlying malignancy, direct suppression of pathogenic autoantibody production through inhibition of B-cell proliferation, or a combination of both mechanisms. Further investigation and long-term follow-up are needed to clarify this issue. This case suggests a possible association between CLL and pemphigus-like autoimmune mucocutaneous disease, but it does not establish a causal link, and any potential benefit of zanubrutinib in this setting remains to be determined. Its value in other forms of pemphigus, however, requires independent investigation.

Several limitations should be noted. First, with respect to the temporal sequence, the patient had already received intravenous and oral corticosteroids before zanubrutinib was initiated, and the later dose increase to 28 mg/day for a cutaneous flare further complicates attribution. Therefore, the improvement of mucosal lesions cannot be separated from the potential contributions of delayed corticosteroid effects, natural fluctuations of CLL, or improved supportive care. As a single-case observation, a direct causal relationship between zanubrutinib and the clinical improvement cannot be established. Second, although the patient exhibited severe mucosal involvement, pemphigus-like lesions, and evidence of CLL, PNP-specific diagnostic tests—including anti-envoplakin and anti-periplakin antibodies—were not performed, and differentiation from pemphigus vulgaris is required in the context of CLL (26). Third, the follow-up period was limited, precluding assessment of the long-term efficacy, safety, and relapse risk associated with Zanubrutinib in CLL-associated PNP. Finally, although chest CT performed in November 2024 did not show imaging features of bronchiolitis obliterans (such as air trapping or mosaic attenuation), pulmonary function testing was not performed; therefore, subclinical small-airway involvement cannot be completely excluded, and regular respiratory surveillance during follow-up is recommended. Future studies with larger sample sizes and multicenter designs are warranted to further evaluate the efficacy, safety, and long-term outcomes of Zanubrutinib in CLL-associated PNP, as well as its potential role in other forms of pemphigus and in individualized treatment strategies.

In addition, the assessment of clinical severity in this report has important limitations. Although approximately 18% body surface area involvement and an oral pain visual analog scale score were documented at baseline, standardized disease activity instruments such as the Pemphigus Disease Area Index (PDAI) or the Autoimmune Bullous Skin Disorder Intensity Score (ABSIS) were not applied. Detailed data—including lesion counts, distribution of oral erosions, degree of functional impairment in oral intake, weight changes, and impact on performance status—were not available in the retrospective records, precluding post‑hoc calculation of these scores. The assessment of clinical improvement therefore relied primarily on available descriptive indicators. In addition, a distinction should be made between the subjective improvement reported by the family and the objective clinical assessment. The improvement described by the family reflects the patient’s subjective experience, including pain relief and resumption of normal oral intake, whereas the clinical assessment of a partial response was based on objective findings—namely, a >50% reduction in erosion area and the absence of new lesions—and does not indicate complete mucosal re‑epithelialization. Although the pre‑ and post‑treatment clinical photographs were taken at the same anatomical sites, the imaging angles and conditions were not entirely standardized, which limits direct visual comparison to some extent.

## Conclusions

In this case, improvements in mucocutaneous lesions and hematological parameters were observed following the initiation of zanubrutinib in the setting of ongoing corticosteroid therapy. This observation may suggest that BTK inhibition and/or control of CLL contributed to the clinical improvement. However, this study does not confirm the efficacy of zanubrutinib in patients with confirmed PNP, nor should its effect be extrapolated to other forms of pemphigus. However, the concurrent corticosteroid uses and CLL control likely contributed to this improvement, making it difficult to isolate the independent effect of zanubrutinib. This case suggests that, in patients with suspected CLL-associated PNP, effective control of the underlying neoplasm may contribute to improvement of mucocutaneous manifestations. Whether zanubrutinib, as a selective BTK inhibitor, could offer a specific therapeutic benefit in CLL-associated PNP through antitumor and/or immunomodulatory mechanisms remains to be determined. Further clinical evidence is needed to evaluate its efficacy and safety.

## Data Availability

The original contributions presented in the study are included in the article/supplementary material. Further inquiries can be directed to the corresponding author.

## References

[1] Lamichhane R Chaudhary S . Delayed diagnosis of pemphigus vulgaris initially presenting as an oral ulcer: a case report. Jnma J Nepal Med Assoc. (2022) 60:641–3. doi: 10.31729/jnma.7594 36705190 PMC9297360

[2] van Beek N Holtsche MM Atefi I Olbrich H Schmitz MJ Pruessmann J . State-of-the-art diagnosis of autoimmune blistering diseases. Front Immunol. (2024) 15:1363032. doi: 10.3389/fimmu.2024.1363032 38903493 PMC11187241

[3] Costan V-V Popa C Hâncu MF Porumb-Andrese E Toader MP . Comprehensive review on the pathophysiology, clinical variants and management of pemphigus (review). Exp Ther Med. (2021) 22:1335. doi: 10.3892/etm.2021.10770 34630689 PMC8495539

[4] Huang S Anderson HJ Lee JB . Paraneoplastic pemphigus/paraneoplastic autoimmune multiorgan syndrome: part II. Diagnosis and management. J Am Acad Dermatol. (2024) 91:13–22. doi: 10.1016/j.jaad.2023.08.084 37714216

[5] Anderson HJ Huang S Lee JB . Paraneoplastic pemphigus/paraneoplastic autoimmune multiorgan syndrome: part I. Clinical overview and pathophysiology. J Am Acad Dermatol. (2024) 91:1–10. doi: 10.1016/j.jaad.2023.08.020 37597771

[6] Adnani BO O’ Brien K Myint ZW Adler BL . Paraneoplastic pemphigus: a striking complication of undiagnosed lymphoma. Case Rep Hematol. (2022) 2022:3641474. doi: 10.1155/2022/3641474 36330223 PMC9626227

[7] Chen C Xu Y Yu J Qian S Xie Y . A first case of successful using of ibrutinib in treating paraneoplastic pemphigus related bronchiolitis obliterans concurrent with CLL. Front Med. (2023) 10:1132535. doi: 10.3389/fmed.2023.1132535 37007770 PMC10056216

[8] Fidder SAR Bolling MC Diercks GFH Pas HH Hooimeijer LHL Bungener LB . Paraneoplastic pemphigus associated with post-transplant lymphoproliferative disorder after small bowel transplantation. Pediatr Transplant. (2021) 25:e14023. doi: 10.1111/petr.14023 34014017

[9] Gao F-Q Zhang J-M Li C-F . Paraneoplastic pemphigus misdiagnosed as juvenile dermatomyositis: a case report. Int J Rheum Dis. (2023) 26:1826–9. doi: 10.1111/1756-185X.14684 37166030

[10] Zhang H Hu B Chen H Wan L Chen J Zhou X . Therapy of paraneoplastic pemphigus with rituximab: a case report. Dermatol Ther. (2021) 34:e15144. doi: 10.1111/dth.15144 34582076

[11] Alatawi Y Alanazi FE Alattar A Alshaman R Ramadan YN Sayad R . Therapeutic impact of zanubrutinib in chronic lymphocytic leukemia: evidence from a systematic review and single-arm meta-analysis. Pharmaceuticals. (2025) 18:1674. doi: 10.3390/ph18111674 41304918 PMC12655045

[12] Xu W Yang S Tam CS Seymour JF Zhou K Opat S . Zanubrutinib monotherapy for naïve and relapsed/refractory chronic lymphocytic leukemia/small lymphocytic lymphoma: a pooled analysis of three studies. Adv Ther. (2022) 39:4251–65. doi: 10.1007/s12325-022-02238-7 35900694

[13] Izutsu K Ishikawa T Shimada K Kubo K Kondo T Fujimoto K . Zanubrutinib in japanese treatment-naive and relapsed/refractory patients with waldenström macroglobulinemia and CLL/SLL. Int J Hematol. (2025) 121:483–93. doi: 10.1007/s12185-025-03925-1 39960615 PMC11922998

[14] Neys SFH Rip J Hendriks RW Corneth OBJ . Bruton’s tyrosine kinase inhibition as an emerging therapy in systemic autoimmune disease. Drugs. (2021) 81:1605–26. doi: 10.1007/s40265-021-01592-0 34609725 PMC8491186

[15] Pulz R Angst D Cenni B . Next generation bruton’s tyrosine kinase inhibitors - characterization of *in vitro* potency and selectivity. Eur J Pharmacol. (2025) 1002:177747. doi: 10.1016/j.ejphar.2025.177747 40403828

[16] De Bondt M Renders J Struyf S Hellings N . Inhibitors of bruton’s tyrosine kinase as emerging therapeutic strategy in autoimmune diseases. Autoimmun Rev. (2024) 23:103532. doi: 10.1016/j.autrev.2024.103532 38521213

[17] García-Sanz R Owen RG Jurczak W Dimopoulos MA McCarthy H Cull G . Outcomes after transition from ibrutinib to zanubrutinib in patients with waldenström macroglobulinemia from the ASPEN study. Blood Adv. (2025) 9:6538–46. doi: 10.1182/bloodadvances.2024015596 40924923 PMC12767813

[18] Rhodes JM Mato AR . Zanubrutinib (BGB-3111), a second-generation selective covalent inhibitor of bruton’s tyrosine kinase and its utility in treating chronic lymphocytic leukemia. Drug Des Devel Ther. (2021) 15:919–26. doi: 10.2147/DDDT.S250823 33688166 PMC7936706

[19] Zou M Zhan K Zhang Y Li L Li J Gao J . A systematic review of paraneoplastic pemphigus and paraneoplastic autoimmune multiorgan syndrome: clinical features and prognostic factors. J Am Acad Dermatol. (2025) 92:307–10. doi: 10.1016/j.jaad.2024.10.013 39426525

[20] Antiga E Bech R Maglie R Genovese G Borradori L Bockle B . S2k guidelines on the management of paraneoplastic pemphigus/paraneoplastic autoimmune multiorgan syndrome initiated by the European academy of dermatology and venereology (EADV). J Eur Acad Dermatol Venereol Jeadv. (2023) 37:1118–34. doi: 10.1111/jdv.18931 36965110 PMC10806824

[21] Pinero-Crespo G Shah N Klager S Kowalewski C . Complete resolution of atypical paraneoplastic pemphigus following treatment with dupilumab. Cureus. (2025) 17:e81033. doi: 10.7759/cureus.81033 40264631 PMC12014160

[22] Ouedraogo E Gottlieb J De Masson A Lepelletier C Jachiet M Salle De Chou C . Risk factors for death and survival in paraneoplastic pemphigus associated with hematologic malignancies in adults. J Am Acad Dermatol. (2019) 80:1544–9. doi: 10.1016/j.jaad.2018.03.043 30981429

[23] Estupiñán HY Berglöf A Zain R Smith CIE . Comparative analysis of BTK inhibitors and mechanisms underlying adverse effects. Front Cell Dev Biol. (2021) 9:630942. doi: 10.3389/fcell.2021.630942 33777941 PMC7991787

[24] Alu A Lei H Han X Wei Y Wei X . BTK inhibitors in the treatment of hematological malignancies and inflammatory diseases: mechanisms and clinical studies. J Hematol Oncolj Hematol Oncol. (2022) 15:138. doi: 10.1186/s13045-022-01353-w 36183125 PMC9526392

[25] Mendes-Bastos P Brasileiro A Kolkhir P Frischbutter S Scheffel J Moñino-Romero S . Bruton’s tyrosine kinase inhibition-an emerging therapeutic strategy in immune-mediated dermatological conditions. Allergy. (2022) 77:2355–66. doi: 10.1111/all.15261 35175630 PMC9545595

[26] Svoboda SA Huang S Liu X Hsu S Motaparthi K . Paraneoplastic pemphigus: revised diagnostic criteria based on literature analysis. J Cutan Pathol. (2021) 48:1133–8. doi: 10.1111/cup.14004 33719070

